# *Coronaviruses*: A patent dataset report for research and development (R&D) analysis

**DOI:** 10.1016/j.dib.2020.105551

**Published:** 2020-04-17

**Authors:** Fiderman Machuca-Martinez, Rubén Camargo Amado, Oscar Gutierrez

**Affiliations:** a Facultad de Ingeniería, Universidad del Valle, Cali, Colombia; b Facultad de Salud, Universidad del Valle, Cali, Colombia

**Keywords:** 2019-nCoV, Patent analysis, Competitive Intelligence, Antiviral Therapy, Triazole, Glycoprotein, Protease inhibitor

## Abstract

This work shows a patent database for *Coronaviruses* that provides an overview of the patenting activity and trends in focused antiviral therapy with the use of triazole based compounds, glycoprotein, and protease inhibitors as possible treatment.

The patent data was obtained from Orbit Intelligence Software using a patent family structure to get a big database that could be used for built patent landscape report (PLR), market analysis, technical and competitive intelligence, and monitoring and survey of a new ideas for the treatment of coronavirus diseases.

The raw data is reported in four databases, which were classified according to different items: legal status (alive, dead), 1^st^ application year (after 2015, 2011-2015, 2006-2010, 2001-2005), and Top 5 International Patents Classifications (IPC).

The main players, the investment trend, markets, geographical distribution, technology overview, technologies distribution, and patent citation are showed by this analysed data report.

Specifications Table

**Table:** 

Subject	Infectious Diseases
Specific subject area	patent landscape report, patent analysis, Bioinformatics
Type of data	Chart Graph Figure
How data were acquired	The data was acquired by Orbit Intelligence Platform
Data format	Raw Analysed
Parameters for data collection	The searching parameters used in Orbit are related to the search equations put into the script: ((CORONAVIRUS)/TI/AB/CLMS/DESC/ODES/OBJ/ADB/ICLM/KEYW AND (ANTIVIRAL THERAPY)/TI/AB/CLMS/DESC/ODES/OBJ/ICLM/KEYW) ((CORONAVIRUS)/TI/AB/CLMS/DESC/ODES/OBJ/ADB/ICLM/KEYW AND (ANTIVIRAL THERAPY)/TI/AB/CLMS/DESC/ODES/OBJ/ICLM/KEYW AND (TRIAZOLE)/TI/AB/CLMS/DESC/ODES/OBJ/ICLM/KEYW) ((CORONAVIRUS)/TI/AB/CLMS/DESC/ODES/OBJ/ADB/ICLM/KEYW AND (ANTIVIRAL THERAPY)/TI/AB/CLMS/DESC/ODES/OBJ/ICLM/KEYW AND (GLYCOPROTEIN)/TI/AB/CLMS/DESC/ODES/OBJ/ICLM/KEYW) ((CORONAVIRUS)/TI/AB/CLMS/DESC/ODES/OBJ/ADB/ICLM/KEYW AND (ANTIVIRAL THERAPY)/TI/AB/CLMS/DESC/ODES/OBJ/ICLM/KEYW AND (PROTEASE INHIBITOR)/TI/AB/CLMS/DESC/ODES/OBJ/ICLM/KEYW) TI: Title; AB: abstract; CLMS: Claims; DESC: description; ODES: Advantages of the Invention Over Previous Art; OBJ: Object of the Invention; ICLM: Independent Claims; KEYW: Keywords; ADB: Concepts
Description of data collection	The raw data consist of four databases, each database has 12 files (XLSX format) and 11 Charts from Orbit Intelligence Platform with the out data profile:Title, Images, Publication numbers, Publication kind codes, Publication dates, Original document, Earliest priority date, Abstract, Inventors, Latest standardized assignees - inventors removed, Representative, Advantages / Previous drawbacks, Independent claims, Object of invention, Technical concepts, Claims, Keywords in context, Technology domains, CPC - Cooperative classification, IPC - International classification, Citing patents - Standardized publication number, Citing patents - Raw information, Cited patents - Standardized publication number, Cited patents - Raw information, Non-Latin cited patents, Cited non-patent literature, Family legal status, Legal status (Pending, Granted, Revoked, Expired, Lapsed), Family legal state, Legal state (Alive, Dead), Legal actions, Independent claims, Dependent claims - Count
Data source location	Institution: Universidad del Valle City/Town/Region: Cali, Valle del Cauca Country: Colombia
Data accessibility	With the article

## Value of the data

• The patent database could be used to determinate new laboratory conditions for preparation, purification, and use for a new treatment of coronaviruses-based disease.
• The patent database can be used to identify trends in the domain of technology for the treatment of the new virus.
• The database can be used for building patent landscape report (PLR).
• The data could help elaborate policies to determine the qualifications for investments in universities, research institutes, foundations, companies, and governments, thus allowing for better decision making in this regard.

## 1 Data Description

The data patents are of high importance because the patents contain technical information about a specific area and they have a high impact on the innovation process [1]. The database consists of two sections:

### 1.1 Raw data

The supporting information section has four databases, each dataset has 12 files (XLXS format) with information selected for specific items and the date of search. Table 1 shows the distribution of data for each search and Table 2 to Table 5 show the information for each file in the database. All files contain information related to: Title, Images, Publication numbers, Publication kind codes, Publication dates, Original document, Earliest priority date, Abstract, Inventors, Latest standardized assignees - inventors removed, Representative, Advantages / Previous drawbacks, Independent claims, Object of invention, Technical concepts, Claims, Keywords in context, Technology domains, CPC - Cooperative classification, IPC - International classification, Citing patents - Standardized publication number, Citing patents - Raw information, Cited patents - Standardized publication number, Cited patents - Raw information, Non-Latin cited patents, Cited non-patent literature, Family legal status, Legal status (Pending, Granted, Revoked, Expired, Lapsed), Family legal state, Legal state (Alive, Dead), Legal actions, Independent claims, Dependent claims - Count.

**Table 1:** Distribution of data for database.

Number database	Name database file	Information
1	CV AV TH	The database contains the information of all patent families related to coronaviruses and antiviral therapy.
2	CV AV TZ	The database contains the information of all patent families related to coronaviruses and antiviral therapy and triazole compounds.
3	CV AV GLYCO	The database contains the information of all patent families related to coronaviruses and antiviral therapy and glycoprotein.
4	CV AV PRO	The database contains the information of all patent families related to coronaviruses and antiviral therapy and protease inhibitors.

**Table 2:** Raw data list for CV AV TH database.

File Number	File name	Information
1	CV AV TH 11-03-2020 Total Patent Families	The file contains the information of all patents. The date of search (11-03-2020). 901 registers
2	CV AV TH 11-03-2020 Alive Patent Families	The file contains the information of alive patents. 572 registers
3	CV AV TH 11-03-2020 Dead Patent Families	The file contains the information of dead patents. 329 registers
4	CV AV TH 11-03-2020 1AY (After 2015) Patent Families	The file contains the information of the patents on 1er application year for 2015-2020 period. 145 registers
5	CV AV TH 11-03-2020 1AY (2011-2015) Patent Families	The file contains the information of the patents on 1er application year for 2011-2015 period. 242 registers
6	CV AV TH 11-03-2020 1AY (2006-2010) Patent Families	The file contains the information of the patents on 1er application year for 2006-2010 period. 245 registers
	CV AV TH 11-03-2020 1AY (2001-2005) Patent Families	The file contains the information of the patents on 1er application year for 2001-2005 period. 123 registers
7	CV AV TH 11-03-2020 IPC A61P-031	A61P-031 = 502 registers A61K-031= 424 registers A61K-039=364 registers C12N-015= 342 registers A61K-038=271 registers
8	CV AV TH 11-03-2020 IPC A61K-031	
9	CV AV TH 11-03-2020 IPC A61K-039	
10	CV AV TH 11-03-2020 IPC C12N-015	
11	CV AV TH 11-03-2020 IPC A61K-038

**Table 3:** Raw data list for CV AV TZ database.

File Number	File name	Information
1	CV AV TZ 11-03-2020 Total Patent Families	The file contains the information of all patents. The date of search (11-03-2020). 170 registers
2	CV AV TZ 11-03-2020 Alive Patent Families	The file contains the information of alive patents. 130 registers
3	CV AV TZ 11-03-2020 Dead Patent Families	The file contains the information of dead patents. 40 registers
4	CV AV TZ 11-03-2020 1AY (After 2015) Patent Families	The file contains the information of the patents on 1er application year for 2015-2020 period. 27 registers
5	CV AV TZ 11-03-2020 1AY (2011-2015) Patent Families	The file contains the information of the patents on 1er application year for 2011-2015 period. 32 registers
6	CV AV TZ 11-03-2020 1AY (2006-2010) Patent Families	The file contains the information of the patents on 1er application year for 2006-2010 period. 59 registers
	CV AV TZ 11-03-2020 1AY (2001-2005) Patent Families	The file contains the information of the patents on 1er application year for 2001-2005 period. 47 registers
7	CV AV TZ 11-03-2020 IPC A61K-031	A61K-031 = 114 registers A61P-031= 109 registers A61P-035=76 registers C12N-015= 75registers A61K-038=71 registers
8	CV AV TZ 11-03-2020 IPC A61P-031	
9	CV AV TZ 11-03-2020 IPCA61P-035	
10	CV AV TZ 11-03-2020 IPC C12N-015	
11	CV AV TZ 11-03-2020 IPC A61K-038

**Table 4:** Raw data list for CV AV GLYCO database.

File Number	File name	Information
1	CV AV GLYCO 11-03-2020 Total Patent Families	The file contains the information of all patents. The date of search (11-03-2020). 513 registers
2	CV AV GLYCO 11-03-2020 Alive Patent Families	The file contains the information of alive patents. 343 registers
3	CV AV GLYCO 11-03-2020 Dead Patent Families	The file contains the information of dead patents. 170 registers
4	CV AV GLYCO 11-03-2020 1AY (After 2015) Patent Families	The file contains the information of the patents on 1er application year for 2015-2020 period. 85 registers
5	CV AV GLYCO 11-03-2020 1AY (2011-2015) Patent Families	The file contains the information of the patents on 1er application year for 2011-2015 period. 142 registers
6	CV AV GLYCO 11-03-2020 1AY (2006-2010) Patent Families	The file contains the information of the patents on 1er application year for 2006-2010 period. 142 registers
	CV AV GLYCO 11-03-2020 1AY (2001-2005) Patent Families	The file contains the information of the patents on 1er application year for 2001-2005 period. 115 registers
7	CV AV GLYCO 11-03-2020 IPC A61P-031	A61P-031 = 286 registers A61K-039= 261 registers A61K-031=233 registers C12N-015= 221registers A61K-038=158 registers
8	CV AV GLYCO 11-03-2020 IPC A61K-039	
9	CV AV GLYCO 11-03-2020 IPC A61K-031	
10	CV AV GLYCO 11-03-2020 IPC C12N-015	
11	CV AV GLYCO 11-03-2020 IPC A61K-038

**Table 5:** Raw data list for CV AV PRO database.

File Number	File name	Information
1	CV AV PRO 11-03-2020 Total Patent Families	The file contains the information of all patents. The date of search (11-03-2020). 367 registers
2	CV AV PRO 11-03-2020 Alive Patent Families	The file contains the information of alive patents. 253 registers
3	CV AV PRO 11-03-2020 Dead Patent Families	The file contains the information of dead patents. 114 registers
4	CV AV PRO 11-03-2020 1AY (After 2015) Patent Families	The file contains the information of the patents on 1er application year for 2015-2020 period. 53 registers
5	CV AV PRO 11-03-2020 1AY (2011-2015) Patent Families	The file contains the information of the patents on 1er application year for 2011-2015 period. 96 registers
6	CV AV PRO 11-03-2020 1AY (2006-2010) Patent Families	The file contains the information of the patents on 1er application year for 2006-2010 period. 108 registers
	CV AV PRO 11-03-2020 1AY (2001-2005) Patent Families	The file contains the information of the patents on 1er application year for 2001-2005 period. 91 registers
7	CV AV PRO 11-03-2020 IPC A61P-031	A61P-031 = 230registers
8	CV AV PRO 11-03-2020 IPC A61K-031	A61K-031= 220registers
9	CV AV PRO 11-03-2020 IPC A61K-038	A61K-038=147registers
10	CV AV PRO 11-03-2020 IPC A61K-039	A61K-039=143 registers
11	CV AV PRO 11-03-2020 IPC C12N-015	C12N-015= 221 registers

### 1.2 Analysed data

This section discloses the processed data from Orbit Intelligence software. The main charts have been selected for each database according to the visualizations recommended by the software. The Figures 1 to 11 show the analysed data for CV AV TH database. The supporting information (SI) has the figures obtained for CV AV TZ, CV AV GLYCO, and CV AV PRO database

**Fig. 1:**
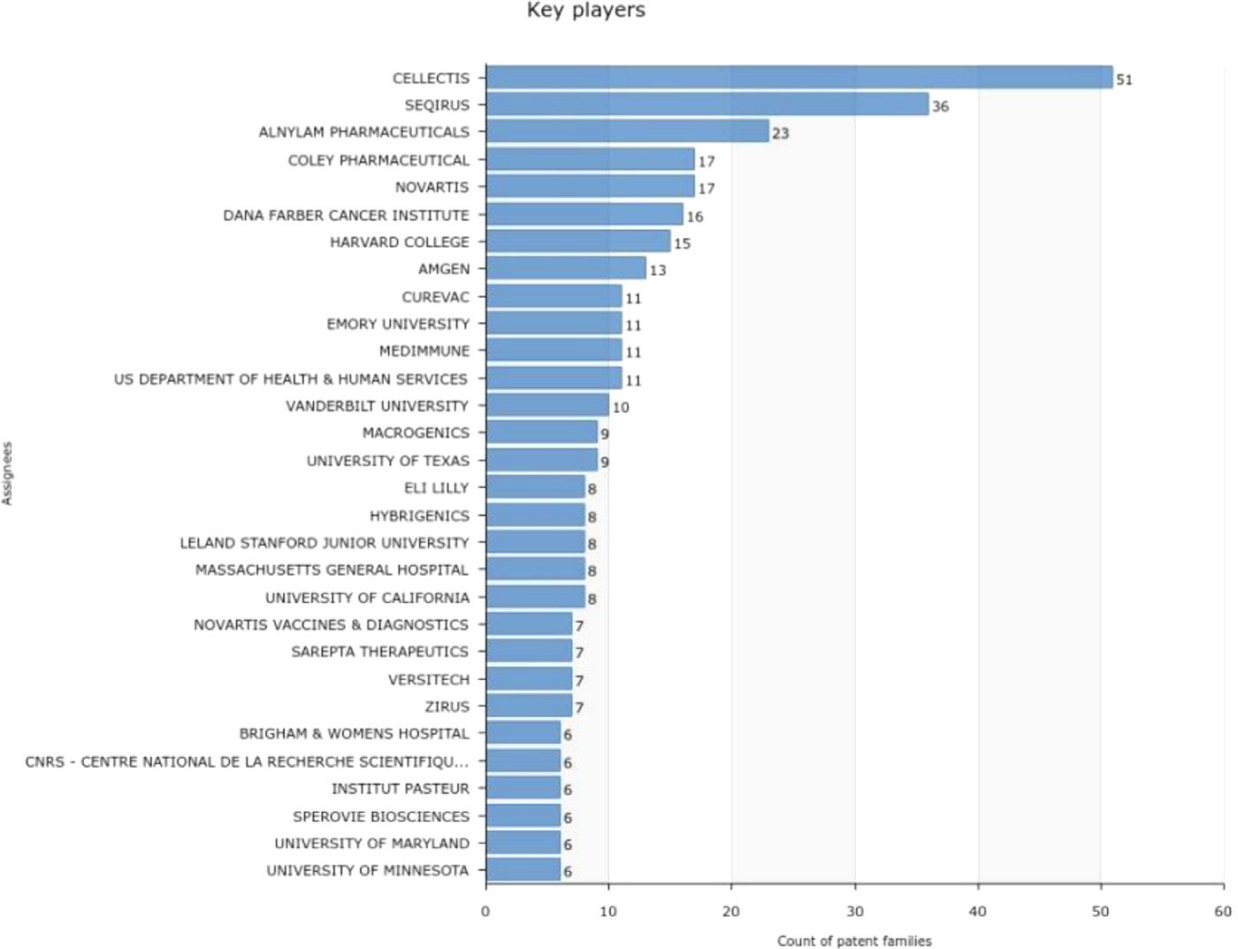
Top applicants according the largest number of patents in the antiviral therapy of coronaviruses.

**Fig. 2:**
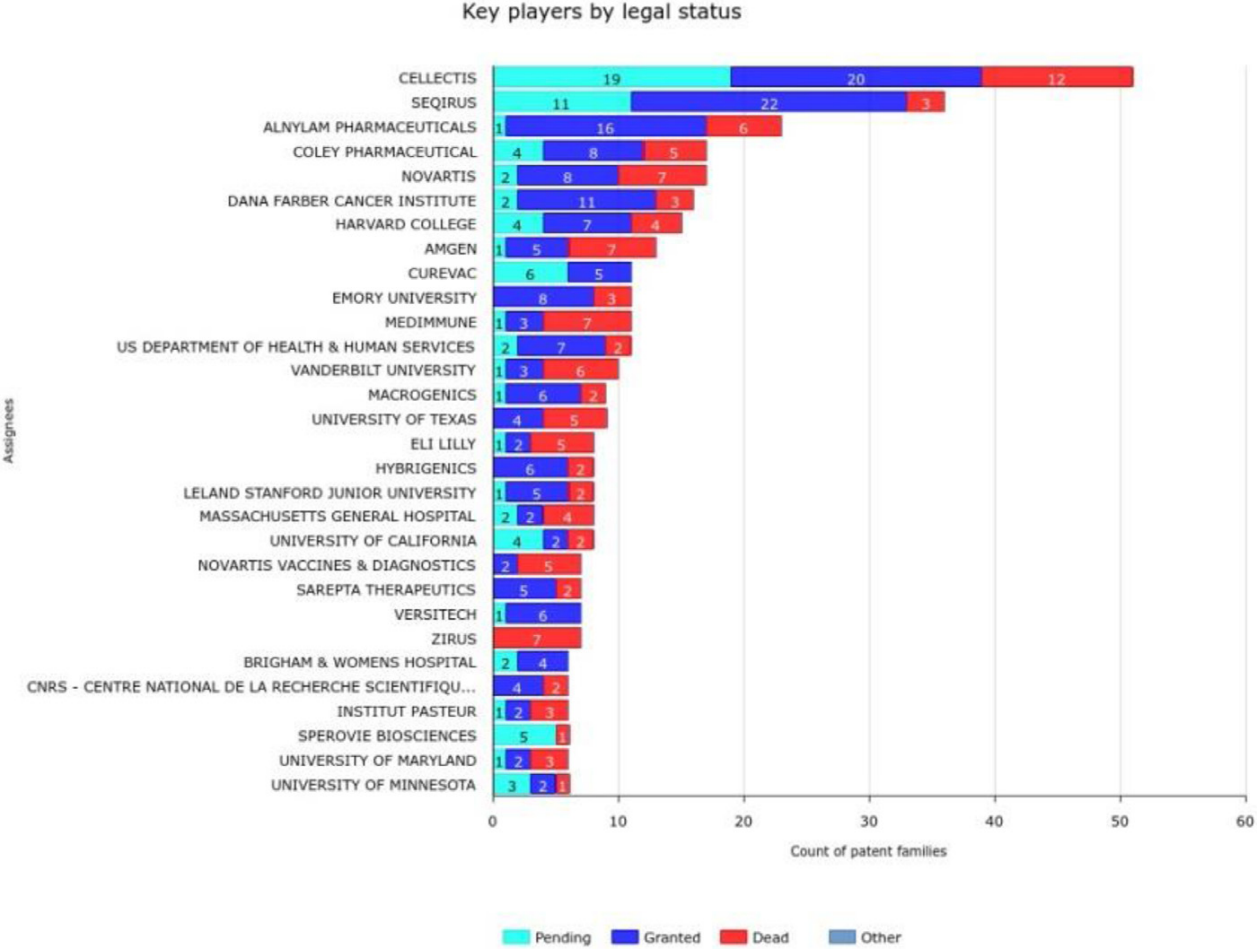
Legal status of patent families for top 30 companies.

**CV AV TH database**, Figure 1 and Figure 2 show the main key players (top 30) according to the size of patents and their legal status (pending, granted, dead) respectively. Also, the figures show the size of the company's portfolios in the antiviral therapy treatment.

**Fig. 3:**
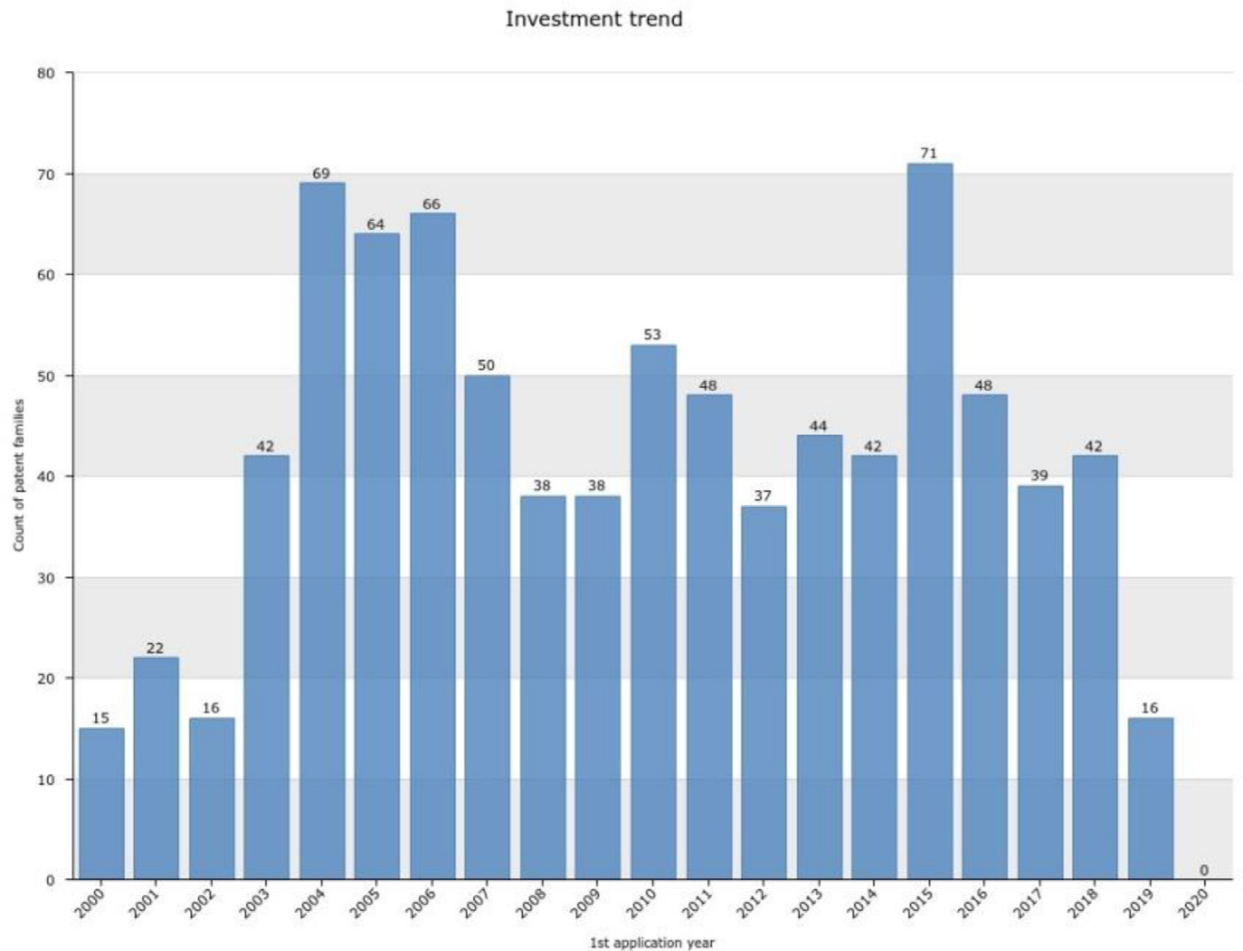
Investment evolution over 20 years.

Figure 3 illustrates the evolution of the investment trend since 2000 to 2019, this data shows he dynamics of inventiveness of the portfolio on patent families.

**Fig. 4:**
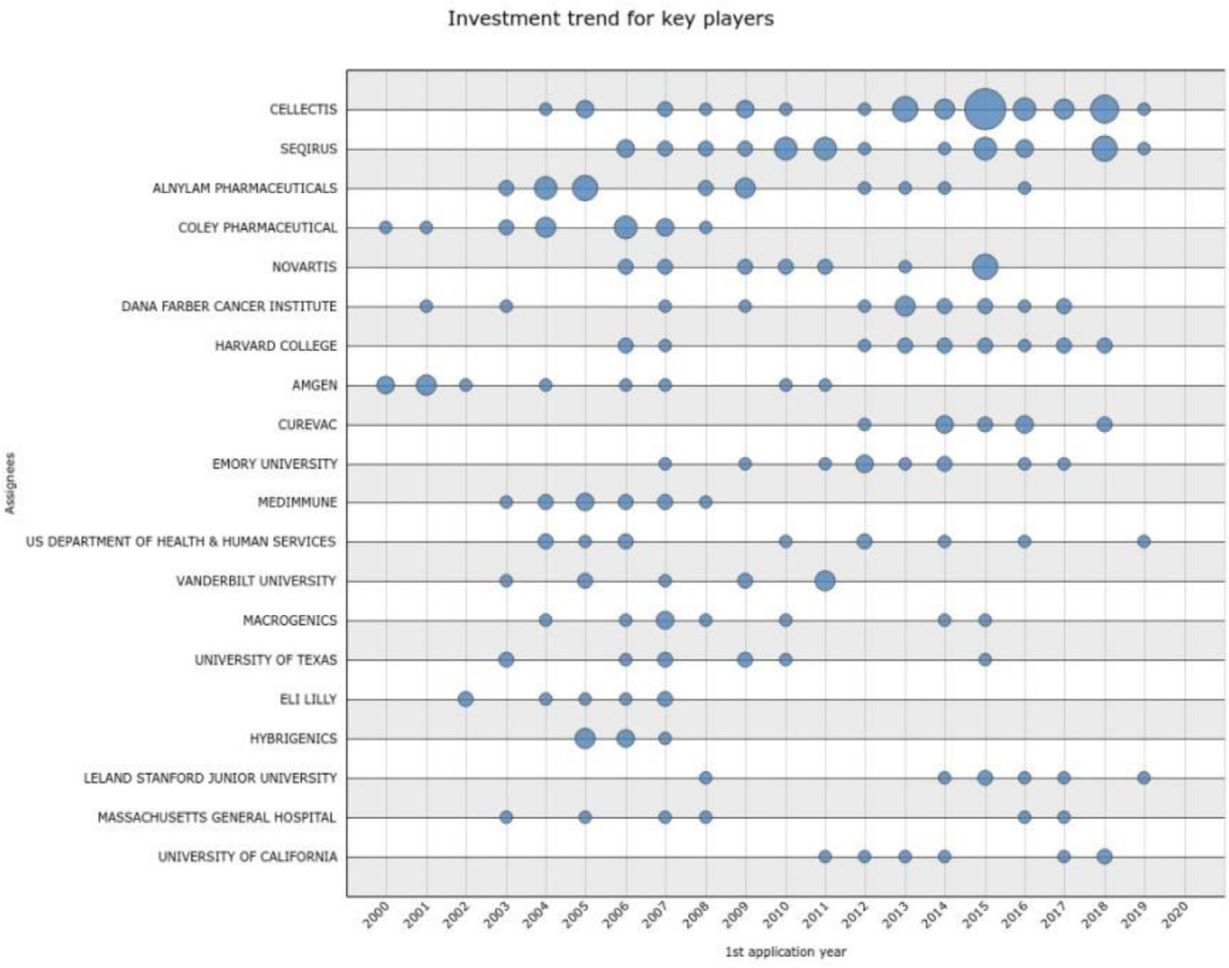
Relative invesment over period (2000-2020) for key players.

Figure 4 shows the trend of applications over time by an applicant and this data is related to the investment (relative size) for company in the time.

**Fig. 5:**
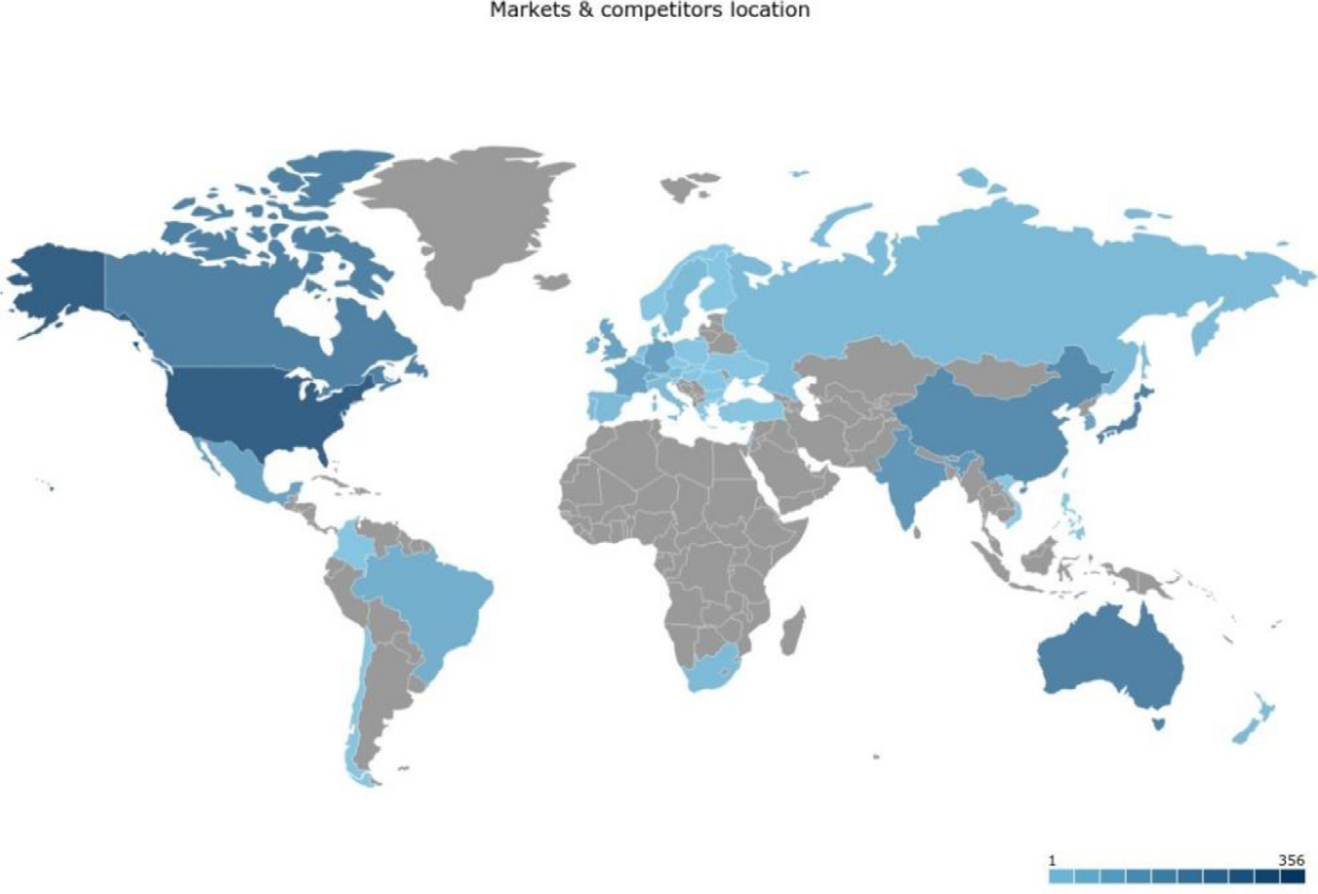
Hot map of possible markets for patent applications.

Figure 5 illustrates the protection map of alive patents in the various national offices.

**Fig. 6:**
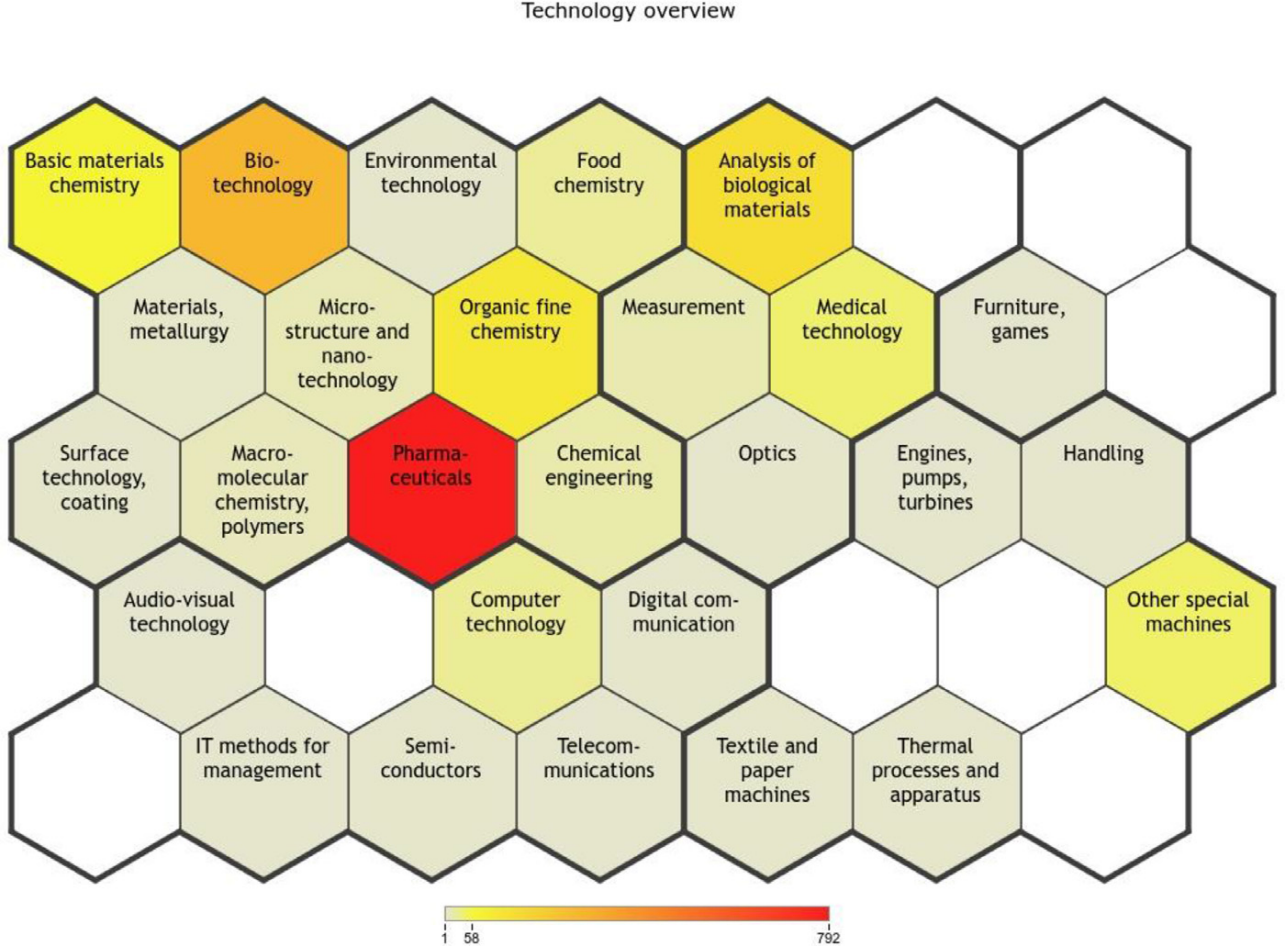
Technological distribution for applications.

**Fig. 7:**
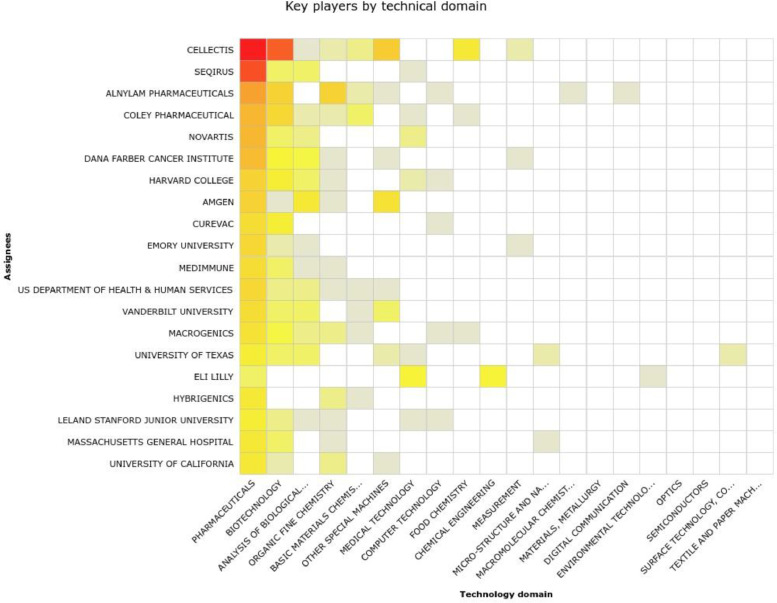
Companies’ distributions by technical profile.

**Fig. 8:**
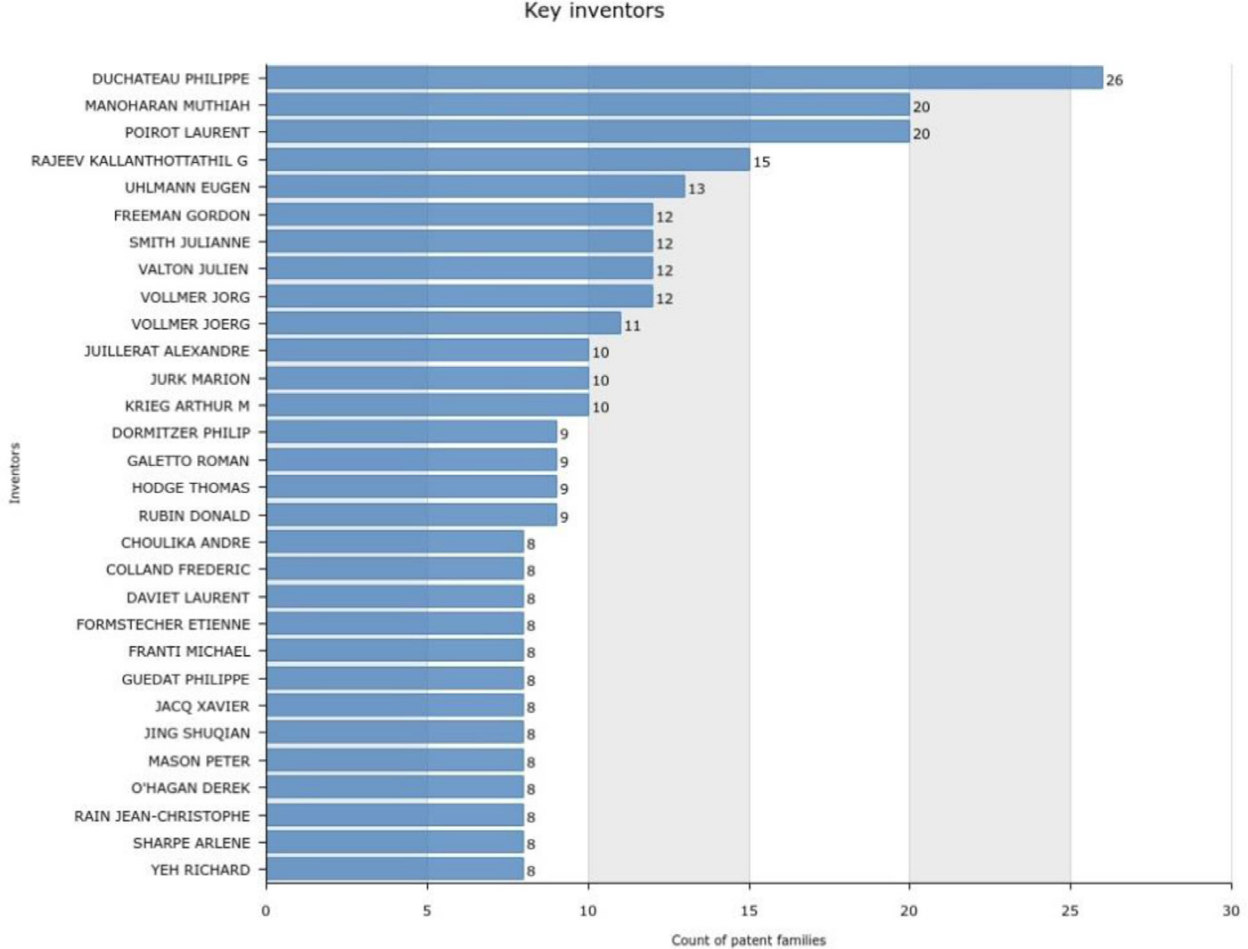
Main inventors in the patent families.

**Fig. 9:**
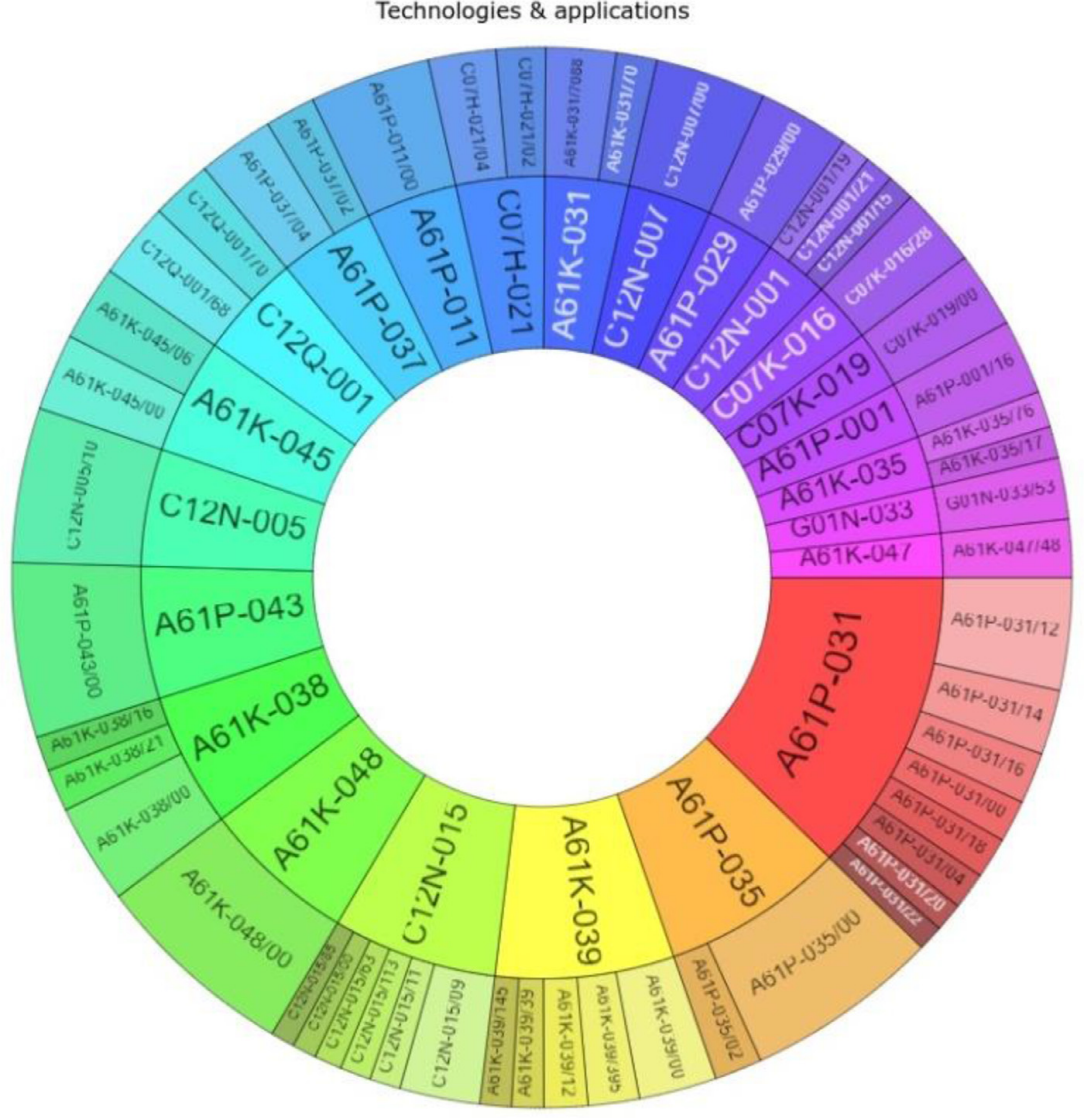
Distribution of main technologies protected by applications area.

Fig. 6, Fig. 7 show heat maps over the domain of technology according to IPC classifications and the distribution of companies. Further, Fig. 8, Fig. 9 establish a relationship between inventors and a technological map based on IPC.

**Fig. 10:**
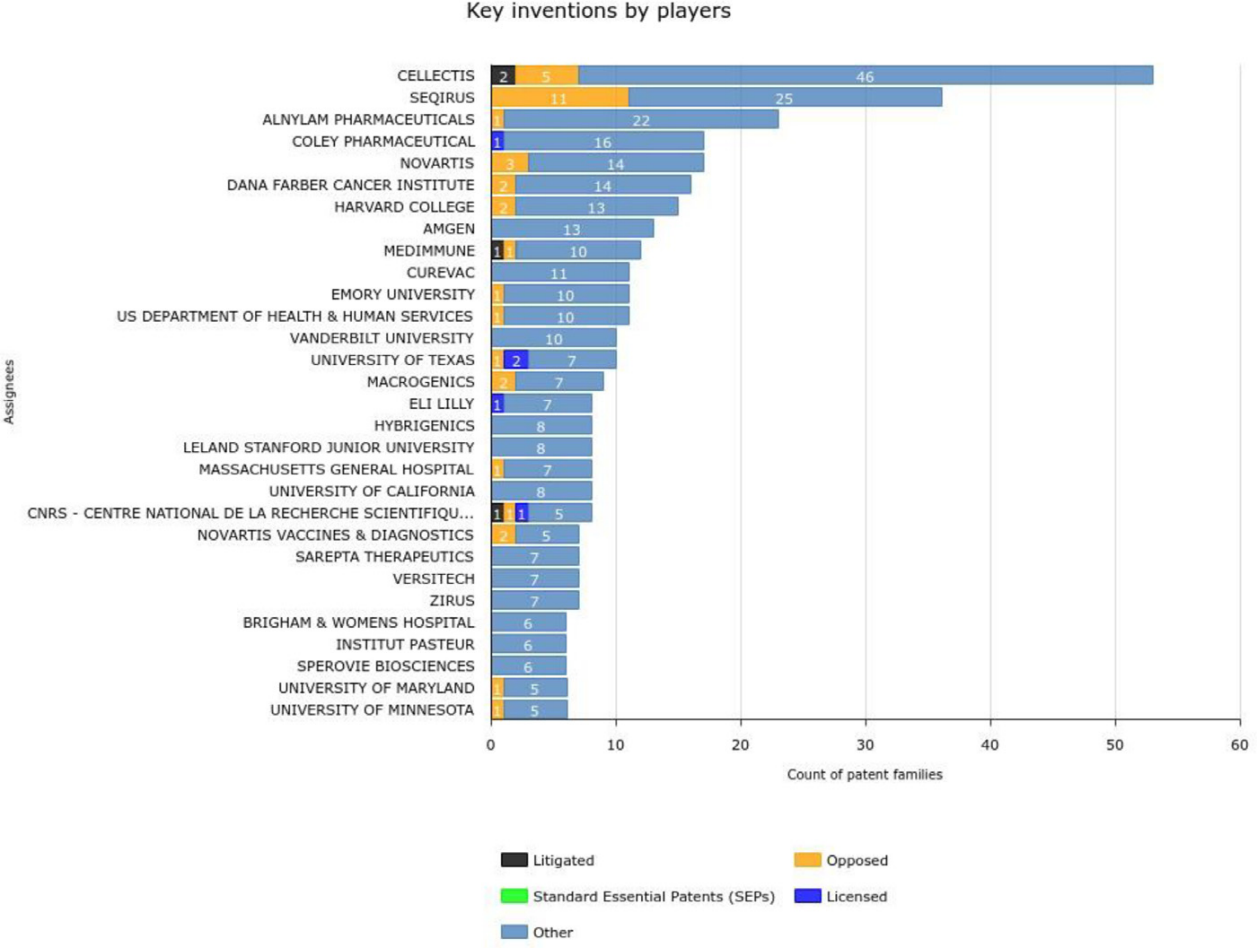
Distribution of key inventions by companies according to legal status.

**Fig. 11:**
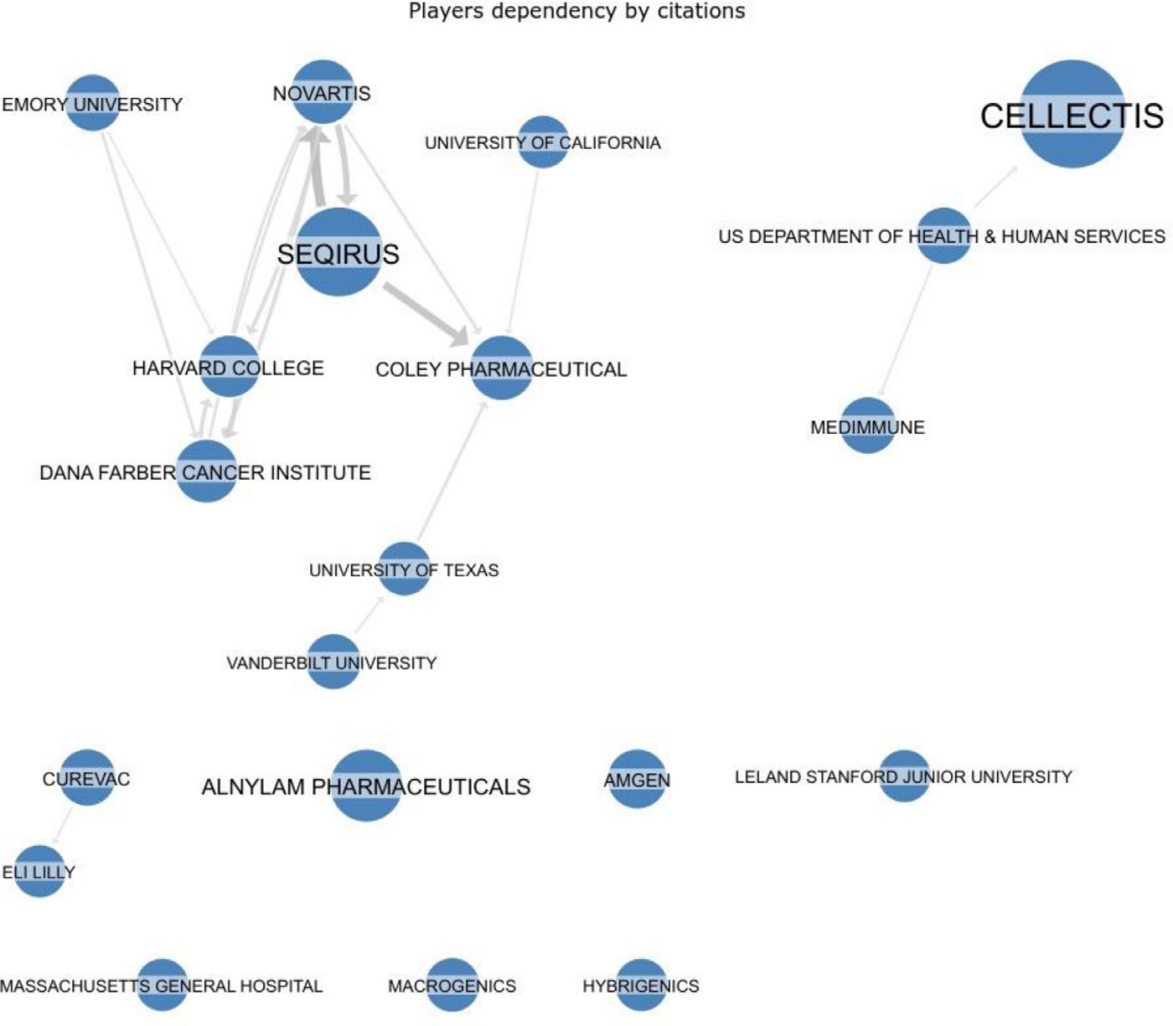
Relationship between companies based on the citations of patents.

Finally, the Fig. 10, Fig. 11 shows the relationship between the key patent families (legal status) and the companies based on the citations.

## 2 Experimental Design, Materials, and Methods

The dataset patents were obtained and analysed using Orbit Intelligence Software (version 1.9.8) from Questel-Orbit.This software has a comprehensive suite for searching, analysing, and managing inventions and IP assets [2,3].

The advanced search option has nine fields: Title, Abstract, Claims, Description, Object of the Invention, Advantages of the Invention Over Previous Art, Independent Claims, Concepts, and Full text. The Fampat Collection was used for search and analysis data and the Fampat module coverage of worldwide patent publications is published by more than 100 patent authorities (Orbit Intelligence Software) [4].

The methodology for searching patents is similar to the one reported by different areas: fisheries [5], petroleum bioremediation techniques [6], propagation in sugar industry [7], analysis of dental caries in primary teeth [8], and hydrogen economic analysis [9].

The selection of keywords was based on a list of compounds that could be useful for the treatment of disease [10], [11], [12], the search strategy was based on the revision of search strategies and then the keywords and IPC combination on the Orbit Platform was used. The advanced search assistant was used with the term related to antiviral therapy. So, the script for the equation search was

((CORONAVIRUS)/TI/AB/CLMS/DESC/ODES/OBJ/ADB/ICLM/KEYW AND (ANTIVIRAL THERAPY)/TI/AB/CLMS/DESC/ODES/OBJ/ICLM/KEYW)
((CORONAVIRUS)/TI/AB/CLMS/DESC/ODES/OBJ/ADB/ICLM/KEYW AND (ANTIVIRAL THERAPY)/TI/AB/CLMS/DESC/ODES/OBJ/ICLM/KEYW AND (TRIAZOLE)/TI/AB/CLMS/DESC/ODES/OBJ/ICLM/KEYW)
((CORONAVIRUS)/TI/AB/CLMS/DESC/ODES/OBJ/ADB/ICLM/KEYW AND (ANTIVIRAL THERAPY)/TI/AB/CLMS/DESC/ODES/OBJ/ICLM/KEYW AND (GLYCOPROTEIN)/TI/AB/CLMS/DESC/ODES/OBJ/ICLM/KEYW)
((CORONAVIRUS)/TI/AB/CLMS/DESC/ODES/OBJ/ADB/ICLM/KEYW AND (ANTIVIRAL THERAPY)/TI/AB/CLMS/DESC/ODES/OBJ/ICLM/KEYW AND (PROTEASE INHIBITOR)/TI/AB/CLMS/DESC/ODES/OBJ/ICLM/KEYW)

Where TI: Title; AB: abstract; CLMS: Claims; DESC: description; ODES: Advantages of the Invention Over Previous Art; OBJ: Object of the Invention; ICLM: Independent Claims; KEYW: Keywords; ADB: Concepts

The raw data were recorded on file (XLXS Format) and the out profile was Title, Images, Publication numbers, Publication kind codes, Publication dates, Original document, Earliest priority date, Abstract, Inventors, Representative, Latest standardized assignees - inventors removed, Advantages / Previous drawbacks, Independent claims, Object of invention, Technical concepts, Claims English, description, Keywords in context, CPC - Cooperative classification, IPC - International classification, and PCL - US patent classification.

The analysis data was made with the IP Business Intelligence module, which is a tool used for decision making. It allows you to analyse big volumes of data and it produces different charts according to the analysis made.

## Appendix Supplementary materials

Image, application 1

Image, application 2
